# Two-year functional outcomes of kinematic versus mechanical alignment in primary total knee arthroplasty: a single-surgeon propensity-matched study in an Asian population

**DOI:** 10.1186/s43019-026-00359-9

**Published:** 2026-11-13

**Authors:** Bo Xiang Bryan Neo, Vishwajit Vijay Patil, Keng-Jin Darren Tay, Hee-Nee Pang, Seng-Jin Yeo, Ming Han Lincoln Liow

**Affiliations:** 1 Yong Loo Lin School of Medicine, National University of Singapore, Singapore, Singapore; 2 Patil Hospital, Maharashtra, India; 3 Department of Orthopaedic Surgery, Singapore General Hospital, Singapore, Singapore

**Keywords:** Kinematic alignment, Mechanical alignment, Propensity score matching, Total knee arthroplasty

## Abstract

**Background:**

Optimal coronal alignment in total knee arthroplasty (TKA) remains controversial, with kinematic alignment (KA) emerging as a patient-specific alternative to conventional mechanical alignment (MA). This study compared 2-year functional outcomes, patient-reported outcome measures (PROMs) and complication rates between KA-TKA and MA-TKA in a propensity score–matched cohort.

**Methods:**

This retrospective single-surgeon study included 1978 consecutive primary TKAs (KA: *n* = 921, 2018–2023; MA: *n* = 1057, 2010–2014). After excluding 76 revision TKAs, 1902 primary TKAs were eligible. A further 441 patients with incomplete follow-up were excluded (KA: *n* = 293, 33.1%; MA: *n* = 148, 14.6%), leaving 1461 patients who underwent propensity score matching. Following 1:1 nearest-neighbour propensity score matching for age, sex, body mass index (BMI) and baseline PROMs, 536 matched pairs (*n* = 1,072) were analysed. Outcomes included range of motion, Knee Society Knee and Function Scores (KSKS, KSFS), Oxford Knee Score (OKS), SF-36, MCID attainment, satisfaction, complications and revision rates at 6 months and 2 years.

**Results:**

Both groups showed substantial postoperative improvement across all outcomes. At 6 months, KA achieved higher postoperative KSKS (86.7 versus 85.3, *p* < 0.001), greater flexion (113.1° versus 110.9°, *p* = 0.010), greater arc of motion (109.8° versus 107.5°, *p* = 0.032) and greater physical functioning, while MA showed greater improvement and higher MCID attainment in SF-36 role physical and bodily pain. Satisfaction at 6 months was higher with KA (95.6% versus 90.8%, *p* = 0.003). At 2 years, KA demonstrated statistically higher KSKS (89.2 versus 86.3, *p* < 0.001), higher KSFS (77.6 versus 75.6, *p* = 0.022), improved range of motion and better physical functioning. Satisfaction remained high and comparable (95.3% versus 95.7%, *p* = 0.838). Complication and revision rates were comparable, with no significant difference in 2-year revision-free survival (99.8% versus 100%, *p* = 0.317).

**Conclusions:**

KA-TKA was associated with statistically higher scores in selected functional domains and comparable MCID attainment, patient satisfaction and revision-free survival at 2 years. These findings suggest KA is a feasible alignment strategy for primary TKA in the short term, although longer-term prospective data are required to confirm these associations.

**Supplementary Information:**

The online version contains supplementary material available at https://doi.org/10.1186/s43019-026-00359-9.

## Introduction

Establishing optimal coronal alignment in total knee arthroplasty (TKA) remains a persistent challenge in knee reconstruction, contributing to patient dissatisfaction in approximately 20% of cases [1]. Alignment philosophy is a major determinant of postoperative patient-reported outcome measures (PROMs). Historically, arthroplasty practice has been polarised between systematic, target-driven strategies and individualized, patient-specific approaches. Mechanical alignment (MA) seeks to restore a neutral coronal limb axis, typically defined as a hip–knee–ankle (HKA) angle of 180° [2]. In contrast, patient-specific paradigms represented by kinematic alignment (KA) aim to reproduce the patient’s native limb and joint line geometry [3]. Intermediate and hybrid strategies have subsequently emerged to reconcile the theoretical advantages and limitations of MA and KA; these include adjusted mechanical alignment (aMA) [4], restricted kinematic alignment (rKA) [5] and functional alignment (FA) [6]. Each philosophy entails distinct principles for bone resection and soft-tissue balancing, with important implications for component positioning, ligament tensioning, biomechanics, implant longevity and clinical outcomes.

MA has long been regarded as the reference standard in total knee arthroplasty, supported by improved PROMs and substantial historical evidence of durable implant survivorship [7, 8]. The MA philosophy standardises bone resection and component positioning to achieve a neutral HKA axis, thereby simplifying surgical technique and facilitating reproducible implant fixation. However, MA inherently disregards considerable inter-individual variability in native coronal alignment and joint-line obliquity [9, 10]. By imposing a uniform limb axis, MA alters native ligament tensioning and joint kinematics, necessitating more frequent soft-tissue releases to achieve balance and potentially disrupting load distribution across the prosthetic surfaces and periprosthetic bone [11–13]. Such alterations have been implicated in non-physiological contact stresses, asymmetric polyethylene wear and aberrant mechanical loading that may compromise long-term implant performance in certain patients.

KA adopts a patient-specific strategy that aims to restore the native joint line, limb alignment and ligament tension, potentially facilitating more physiological knee kinematics and reducing the need for soft-tissue releases [14]. Early studies suggest that KA may improve early functional recovery, range of motion and patient-perceived naturalness [14]. However, most comparative evidence derives from predominantly Western populations, limiting its generalisability to Asian cohorts. This is potentially important given the higher prevalence of constitutional varus, population-specific knee morphology and greater functional demands for deep flexion associated with squatting and kneeling in many Asian populations [15]. These differences may influence the relative performance of alignment strategies and warrant further evaluation in Asian populations.

This large-scale, single-surgeon study therefore aimed to compare functional outcomes, patient-reported outcome measures and safety outcomes between mechanical and kinematic alignment in primary TKA within an Asian population, with particular focus on early- to mid-term recovery, range of motion and revision rates.

## Materials and methods

### Study design and patient selection

This retrospective propensity score-matched cohort study compared outcomes following conventional primary total knee arthroplasty (TKA) performed using mechanical alignment (MA) and kinematic alignment (KA) by a single fellowship-trained surgeon. MA TKAs were performed between 2010 and 2014, whereas KA TKAs were performed between 2018 and 2023. Ethical approval was obtained from the Centralised Institutional Review Board and Ethics Committee prior to study initiation (CIRB Ref: 2024/4046), and the study was conducted in accordance with the Declaration of Helsinki. Patient data were extracted from the institutional TKA registry and anonymised prior to analysis.

**Fig. 1:**
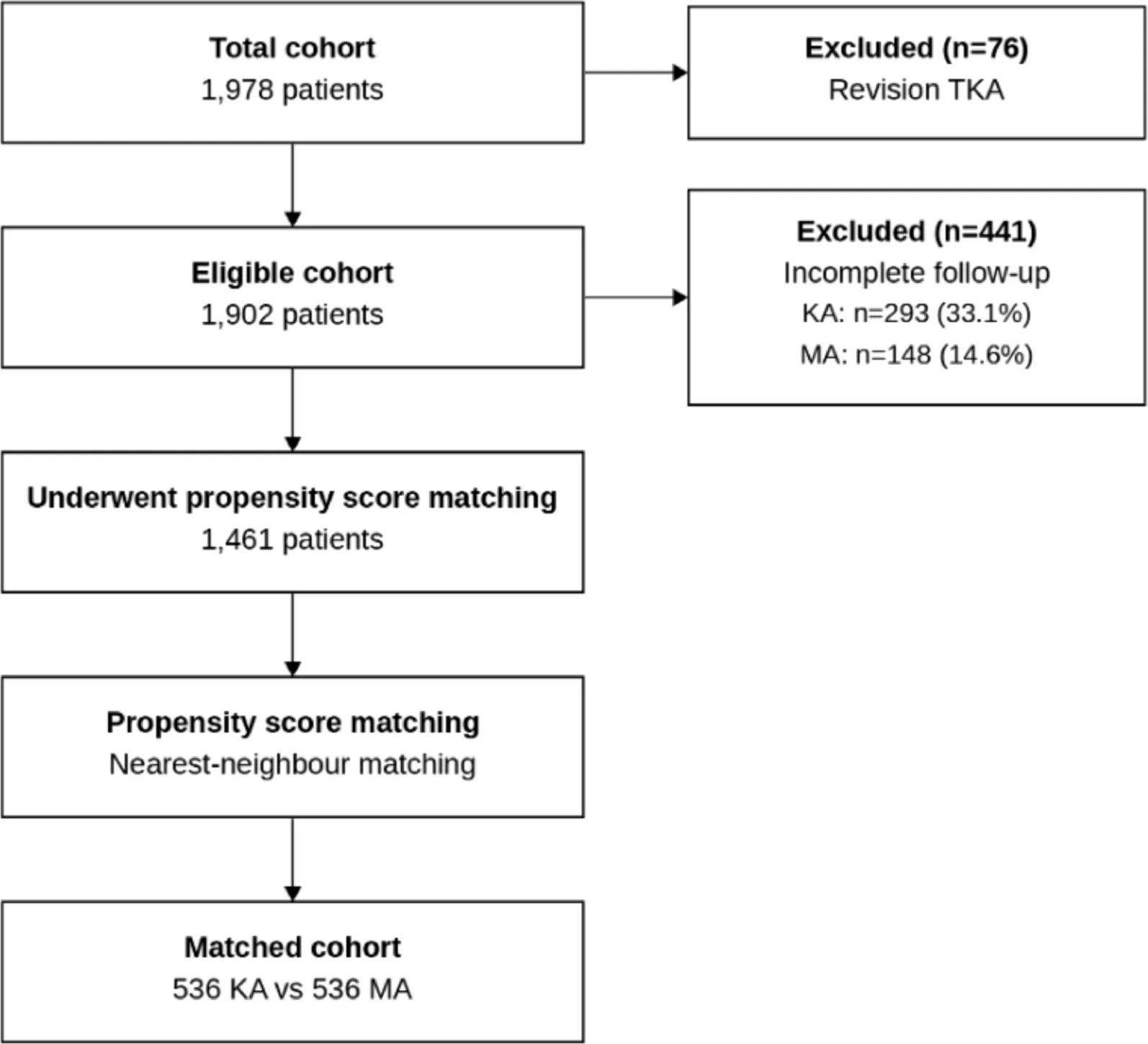
Cohort selection and propensity score matching

A total of 1978 patients who underwent conventional TKA were initially identified. Patients who underwent revision TKA were excluded (*n* = 76), yielding an eligible cohort of 1902 patients. Patients with incomplete follow-up data at 6 months or 2 years were subsequently excluded (*n* = 441). The cohort selection process and propensity score matching are illustrated in Fig. 1. Of the 1902 patients remaining after exclusion of revision TKAs, 293 of 885 KA patients (33.1%) and 148 of 1017 MA patients (14.6%) were excluded due to incomplete follow-up at 6 months or 2 years. To minimise selection bias and account for baseline differences between alignment strategies, propensity score matching was performed using 1:1 nearest-neighbour matching based on age, sex, demographic variables and preoperative functional scores. Following matching, 536 patients in the KA group were matched to 536 patients in the MA group, resulting in a final study cohort of 1072 patients (all standardised mean differences < 0.1 after matching). This sample size met the requirements established by a pre-study power analysis. A priori power analysis was conducted based on the MCID of the OKS and standard deviation reported by Clement et al., yielding an effect size of 0.82 [16]. To achieve 90% power with an alpha level of 0.05, a minimum of 27 patients per group was required to detect a significant difference between independent means. A separate power analysis in a South-East Asian population by Liow et al. indicated that 29 knees per group were sufficient to detect clinically relevant differences between TKA cohorts [17].

Demographic variables recorded included age, sex, race, height and body mass index (BMI). Patient-reported and clinician-assessed outcome measures collected preoperatively and postoperatively included the Knee Society Knee Score (KSKS), Knee Society Function Score (KSFS), Oxford Knee Score (OKS), the Short Form-36 (SF-36), as well as satisfaction scores [16]. The Oxford Knee Score (OKS) was analysed using the original scoring convention, whereby lower scores indicate better knee function and higher scores indicate worse function [18]. Change in OKS was calculated as baseline score minus postoperative score, such that a positive change represented improvement attainment was assessed based on this change-score direction. Patient satisfaction was assessed using a 6-point Likert scale ranging from 1 (excellent) to 6 (terrible). Responses were dichotomized, with scores of 1–3 classified as ‘satisfied’ and scores of 4–6 classified as ‘not satisfied’ [19].

### Surgical technique

All patients underwent the standard medial parapatellar approach and received a cruciate-retaining, fixed bearing cemented total knee system (P.F.C.® Total Knee System, DePuy Synthes, Massachusetts, USA).

#### Mechanical alignment technique

Femoral alignment was established using an intramedullary rod inserted approximately 7–10 mm anterior to the posterior cruciate ligament origin, with the valgus correction angle set between 5° and 7° according to preoperative long-leg radiographic planning. Distal femoral resection involved removal of at least 9 mm of bone from the more prominent femoral condyle. Tibial preparation was performed using an extramedullary alignment jig centred over the medial third of the tibial tubercle and aligned distally with the transmalleolar axis. The tibial posterior slope was fixed at 5°, and the resection depth was referenced from the tibial plateau using a stylus. Flexion and extension gaps were balanced with spacer blocks to achieve a rectangular gap configuration. Femoral component rotation was subsequently set at 3° of external rotation relative to the posterior condylar axis, followed by chamfer cuts and patellar resurfacing to restore native patellar thickness. After satisfactory trialling, the definitive implants were cemented in place and standard layered wound closure was performed.

#### Kinematic alignment technique

In the kinematic alignment total knee arthroplasty (KA-TKA) group, patients underwent routine KA-TKA as described by Stephen M. Howell [3]. A standard medial parapatellar approach was similarly used in all cases. Following exposure of the joint, residual intact cartilage on the distal femur was removed down to subchondral bone. Distal femoral resection was performed using the femoral cutting guide while accounting for approximately 2 mm of cartilage wear and 1 mm sawblade thickness to reproduce the 9 mm implant thickness, resulting in a planned 6 mm resection from both the medial and lateral femoral condyles, confirmed using calipers. The posterior referencing guide was positioned at 0° rotation relative to the posterior condyles. In cases with tibial bone loss, a line parallel to the native posterior condylar axis was marked on the tibia to guide the varus–valgus orientation of the tibial cut. Proximal tibial resection was then performed using a conventional extramedullary guide and verified with caliper measurements. The posterior tibial slope was adjusted to reproduce the native anatomy, with approximately 8 mm of resection from the lateral tibial plateau. The objective was to achieve equivalent medial and lateral tibial resections in the anteroposterior plane, with intraoperative adjustments made where required. All patellae were resurfaced using an onlay prosthesis. Final femoral sizing was then completed, followed by use of the appropriate 4-in-1 cutting block to complete the femoral resections. Following satisfactory trial implantation, the definitive components were cemented and standard layered closure was performed.

Postoperative care followed a standardised institutional protocol for all patients. This protocol included same-day mobilisation with physiotherapy initiated on postoperative day 0 and a multimodal, opioid-sparing analgesic regimen. Analgesia consisted of paracetamol and non-steroidal anti-inflammatory drugs (NSAIDs) when not contraindicated. The protocol was applied uniformly across all study participants.

### Data collection

Demographic data such as age, sex and body mass index were recorded. Patients were assessed by independent trained research coordinators and physiotherapists at the institution’s Orthopaedic Diagnostic Centre (ODC), who were not involved in the surgical care of the patients and were blinded to alignment group allocation. Data measured included active range of motion (flexion and extension) and PROMs such as the Knee Society Knee (KSKS) and Function Scores (KSFS), the Oxford Knee Score (OKS) and the Short Form-36 (SF-36) at 6 months and 2 years postoperatively. The proportion of patients achieving the minimal clinically important difference (MCID) at 6 months was computed for both the MA and KA cohorts. These MCID thresholds were derived from established values in the existing literature [20–22]. Coronal alignment was assessed pre- and postoperatively using a standardised goniometer protocol by independent trained assessors at the Orthopaedic Diagnostic Centre. Knee varus/valgus alignment readings were measured using a goniometer by a trained physiotherapist in our institution. Patients were first instructed to stand barefoot with toes pointing forward. The centre location of the patella and ankle were marked out with a pen. The centre of the goniometer was then aligned and placed on the centre of the patella, with one arm of the goniometer extending along the midline of the thigh and the other arm extending along the axis of the lower leg towards the centre of the ankle. The goniometer reading was then obtained to the nearest 1°. Given that the angle between the anatomic axis and mechanical axis of the femur is approximately 5–7°, we considered neutral alignment to be 175° (5° valgus) [23]. Clinical goniometry has been shown to correlate closely with radiographic HKA measurement in the arthroplasty setting [24].

### Statistical analysis

Continuous variables were assessed for normality using the Shapiro–Wilk test. Normally distributed data were presented as mean ± standard deviation (SD), while categorical variables were expressed as frequencies and percentages. Comparisons between groups were performed using the independent-samples *t*-test for normally distributed continuous variables and the Mann–Whitney *U* test for non-parametric variables. Categorical variables were compared using the chi-square test or Fisher’s exact test where appropriate. Statistical significance was defined as a two-tailed *p* value < 0.05. To mitigate treatment selection bias, propensity score matching was employed. Cohen’s *d* is reported for baseline continuous variables as a standardised effect size to support comparability between groups independent of sample size; |*d*| < 0.2 is conventionally considered a negligible difference [25]. Propensity scores were estimated via L2-regularised logistic regression, incorporating age, sex, BMI and all available preoperative patient-reported outcome measures (KSKS, KSFS, OKS, PCS, MCS and all eight SF-36 subscale scores) as covariates. Preoperative range of motion (flexion, extension and arc of motion) was not included as a covariate in the propensity score model, however, post-matching standardised mean differences confirmed excellent balance in preoperative ROM between groups. Regularisation was applied to address collinearity between summary (PCS, MCS) and subscale SF-36 scores. Optimal 1:1 nearest-neighbour matching without replacement was performed using a caliper of 0.2 standard deviations of the logit propensity score. Covariate balance was assessed using standardised mean differences (SMDs); all SMDs were < 0.10 after matching, confirming adequate balance (Table 2). Given that the matched cohort (*n* = 536/group) substantially exceeded the sample size required by the a priori power calculation, Cohen’s *d* with 95% confidence intervals was additionally calculated for all continuous postoperative outcomes (Table 3, Table 4) to contextualise statistical findings against clinically meaningful effect size thresholds, rather than relying on *p*-values alone. All statistical analyses were performed using IBM SPSS Statistics version 27.0 (IBM Corp., Armonk, NY, USA). Revision-free survival to 2 years was estimated using the Kaplan–Meier method and groups compared using the log-rank test. For the purposes of Kaplan–Meier analysis, revision was defined as any return to theatre for exchange, removal or replacement of a prosthetic component. Manipulation under anaesthesia, arthrotomy and washout for infection and fracture fixation without component exchange were not counted as revision events in the survival analysis (Table 1).

**Table 1:** Inclusion and exclusion criteria

Inclusion criteria	Exclusion criteria
Patients undergoing primary TKA	Patients undergoing revision TKA
Patients undergoing TKA for primary end-stage knee OA	Patients undergoing TKA for indications other than primary knee OA (e.g. inflammatory arthritis)
Use of conventional instrumentation systems	Use of robotic systems
Documentation of early outcome data (e.g. Surgical Duration, post-operative pain scores, LOS and ambulation distance) and pre-operative and 6 months functional outcomes (e.g. KSS, SF-36)	Missing early outcome data, pre-operative, 6 months and 2 years functional data (e.g. from lost-to-follow-up)

## Results

### Demographics

A total of 1461 primary TKAs underwent propensity score matching. After 1:1 nearest-neighbour propensity matching based on age, sex, BMI and baseline PROMs, 536 well-balanced pairs were obtained. Before matching, differences were observed in age and several baseline SF-36 domains. Propensity score matching yielded two well-balanced cohorts of 536 patients each, with all matched variables demonstrating negligible effect sizes (Cohen’s *d* < 0.10) and no statistically significant between-group differences (*p* > 0.05), confirming adequate covariate balance prior to outcome analysis. The matched cohorts had comparable baseline demographics, PROMs and ROM (Table 2). Pre- and postoperative coronal alignment was assessed using a standardised goniometer protocol by independent trained assessors. Preoperatively, both groups were predominantly varus (KA: 72.2%, MA: 75.3%, *p* = 0.279), with a small but statistically significant difference in mean tibiofemoral angle (KA: 5.8° ± 4.3° versus MA: 6.5° ± 4.5°, *p* = 0.017, *d* = 0.16). Postoperative tibiofemoral angles also differed modestly between groups (KA: 4.5° ± 2.1° versus MA: 4.2° ± 1.9°, *p* = 0.030, *d* = 0.15), with both cohorts demonstrating correction to predominantly valgus alignment (KA: 92.4%, MA: 94.4%, *p* = 0.244). These goniometric data provide supportive clinical evidence that both alignment strategies achieved coronal correction in this cohort, though definitive confirmation of component-level alignment, joint-line orientation and residual constitutional alignment requires formal long-leg radiographic assessment, which represents a limitation of the present study. It should be noted that goniometric measurement captures the anatomic tibiofemoral angle, which incorporates the natural valgus offset between the anatomic and mechanical femoral axes of approximately 5–7° [23]. Accordingly, a knee restored to its native constitutional alignment by unrestricted calipered KA would be expected to read as valgus on goniometry even where the mechanical hip–knee–ankle axis remains in slight varus, consistent with the intended KA target. The high rate of postoperative valgus alignment on goniometry (92.4% in the KA group) therefore does not imply mechanical overcorrection and should be interpreted in this context.

**Table 2:** Baseline demographics

	KA	MA	*p*-value	Cohen’s *d*
Age (years)	68.6 ± 7.3	68.8 ± 7.0	0.453	−0.03
BMI (kg/m^2^)	27.3 ± 4.6	27.1 ± 4.1	0.906	0.02
Female sex (%)	76.1%	77.1%	0.773	—
Flexion (°)	116.6 ± 17.7	117.4 ± 17.5	0.328	−0.05
Extension (°)	6.8 ± 7.0	7.5 ± 7.6	0.204	−0.09
Range of motion (°)	109.8 ± 21.2	110.0 ± 20.2	0.827	−0.01
Preop Knee Society Knee Score (KSKS)	37.2 ± 17.3	36.7 ± 17.1	0.647	0.03
Preop Knee Society Function Score (KSFS)	53.4 ± 18.2	52.7 ± 17.6	0.641	0.04
Preop Oxford Knee Score (OKS)*	35.0 ± 7.9	35.3 ± 7.8	0.544	−0.05
Preop SF-36 Physical Function	41.3 ± 25.2	39.3 ± 22.1	0.289	0.08
Preop SF-36 Role Physical	18.5 ± 32.5	16.2 ± 30.2	0.242	0.07
Preop SF-36 Bodily Pain	33.4 ± 17.7	32.5 ± 16.2	0.784	0.06
Preop SF-36 General Health	68.7 ± 20.7	68.0 ± 20.3	0.576	0.03
Preop SF-36 Vitality	71.1 ± 21.9	70.6 ± 21.1	0.632	0.02
Preop SF-36 Social Function	55.9 ± 35.7	52.7 ± 35.3	0.103	0.09
Preop SF-36 Role Emotional	91.8 ± 26.6	91.3 ± 27.3	0.682	0.02
Preop SF-36 Mental Health	80.7 ± 17.3	80.1 ± 17.3	0.535	0.04
Preop SF-36 Physical Component Summary (PCS)	32.9 ± 9.2	32.1 ± 8.3	0.268	0.09
Preop SF-36 Mental Component Summary (MCS)	52.4 ± 11.4	51.9 ± 11.1	0.325	0.04

^*^Oxford Knee Score (OKS) outcomes according to the original scoring convention, with scores ranging from 12 to 60, where lower scores indicate better knee function. Positive change scores represent improvement and were calculated as baseline OKS minus postoperative OKS

### 6-Month postoperative outcomes

At 6 months, both KA and MA groups demonstrated substantial postoperative improvements across all PROMs. KA achieved significantly higher postoperative Knee Society Knee Scores (KSKS) (86.7 versus 85.3, *p* < 0.001, *d* = 0.14, 95% CI 0.02 to 0.27), greater end-range flexion (113.1° versus 110.9°, *p* = 0.010, *d* = 0.15, 95% CI 0.03 to 0.27) and greater arc of motion (109.8° versus 107.5°, *p* = 0.032, *d* = 0.13, 95% CI 0.01 to 0.26), while extension was comparable between groups (*d* = −0.01, 95% CI −0.13 to 0.11). Postoperative Knee Society Function Scores (KSFS) (*d* = 0.04, 95% CI −0.08 to 0.16), Oxford Knee Scores (OKS) (*d* = 0.08, 95% CI −0.04 to 0.20) and their respective change-from-baseline scores (KSFS change *d* = −0.03, 95% CI −0.15 to 0.09; OKS change *d* = −0.12, 95% CI −0.24 to −0.00) were comparable between groups, with no significant differences in MCID attainment. All effect sizes for these comparisons were negligible (|*d*| < 0.15).

For SF-36 domains, KA demonstrated significantly higher physical functioning scores at 6 months (70.3 versus 67.6, *p* < 0.001, *d* = 0.11, 95% CI −0.01 to 0.23). In contrast, MA showed greater improvement in role physical (54.1 versus 48.3, *p* = 0.040, *d* = −0.16, 95% CI −0.28 to −0.04) and bodily pain (36.8 versus 33.2, *p* = 0.023, *d* = −0.14, 95% CI −0.26 to −0.02), with higher MCID attainment for both domains (role physical: 75.7% versus 69.1%, *p* = 0.018; bodily pain: 78.7% versus 72.9%, *p* = 0.031). All other SF-36 domains, including physical component summary (PCS) (*d* = −0.03, 95% CI −0.15 to 0.09) and mental component summary (MCS) (*d* = −0.02, 95% CI −0.14 to 0.10), were comparable between cohorts. Patient satisfaction was high in both groups but significantly greater in the KA group (95.6% versus 90.8%, *p* = 0.003) (Table 3). Secondary SF-36 subscale outcomes at 6 months are reported in Supplementary Table 1.

**Table 3:** 6-Month PROMs and ROM outcomes

	KA	MA	*p*-value	Cohen’s *d* (95% CI)
*Knee Society Knee Score (KSKS)*				
Score at 6 months	86.7 ± 12.9	85.3 ± 11.1	< 0.001	0.14 (0.02 to 0.27)
Change from baseline	49.6 ± 20.9	48.6 ± 19.7	0.244	0.06 (−0.06 to 0.18)
MCID attained (≥ 6.4), %	96.7%	97.1%	0.814	—
*Knee Society Function Score (KSFS)*				
Score at 6 months	72.4 ± 18.4	71.4 ± 17.0	0.229	0.04 (−0.08 to 0.16)
Change from baseline	18.9 ± 20.6	18.6 ± 19.2	0.876	0.03 (−0.09 to 0.15)
MCID attained (≥ 5.9), %	70.3%	74.6%	0.137	—
Oxford Knee Score (OKS)^*^				
Score at 6 months	19.5 ± 5.5	19.0 ± 4.5	0.649	0.08 (−0.04 to 0.20)
Change from baseline	15.4 ± 8.4	16.3 ± 8.0	0.109	−0.12 (−0.24 to −0.00)
MCID attained (≥ 5.0), %	90.8%	93.5%	0.135	—
*SF-36 Physical Function*				
Score at 6 months	70.3 ± 23.5	67.6 ± 20.0	< 0.001	0.11 (−0.01 to 0.23)
Change from baseline	29.1 ± 27.9	28.3 ± 23.6	0.611	0.00 (−0.12 to 0.12)
MCID attained (≥ 11.6), %	72.5%	75.0%	0.392	—
*SF-36 Role Physical*				
Score at 6 months	66.9 ± 39.8	70.3 ± 37.8	0.257	−0.11 (−0.23 to 0.01)
Change from baseline	48.3 ± 46.6	54.1 ± 44.5	0.040	−0.16 (−0.28 to −0.04)
MCID attained (≥ 11.7), %	69.1%	75.7%	0.018	—
*SF-36 Bodily Pain*				
Score at 6 months	66.6 ± 26.4	69.3 ± 23.7	0.114	−0.12 (−0.24 to 0.01)
Change from baseline	33.2 ± 28.6	36.8 ± 26.2	0.023	−0.14 (−0.26 to −0.02)
MCID attained (≥ 16.9), %	72.9%	78.7%	0.031	—
*SF-36 Physical Component Summary (PCS)*				
Score at 6 months	47.0 ± 9.6	47.2 ± 9.2	0.588	−0.03 (−0.15 to 0.09)
Change from baseline	14.2 ± 11.9	15.1 ± 10.7	0.308	−0.10 (−0.22 to 0.02)
MCID attained (≥ 10.0), %	64.2%	69.4%	0.083	—
*SF-36 Mental Component Summary (MCS)*				
Score at 6 months	56.5 ± 9.5	56.8 ± 9.6	0.543	−0.02 (−0.14 to 0.10)
Change from baseline	4.1 ± 11.1	4.9 ± 11.0	0.083	−0.07 (−0.20 to 0.05)
MCID attained (≥ 10.0), %	26.4%	28.2%	0.553	—
*Range of motion*				
Flexion at 6 months	113.1 ± 13.4	110.9 ± 13.7	0.010	0.15 (0.03 to 0.27)
Extension at 6 months	3.3 ± 4.4	3.3 ± 4.7	0.666	−0.01 (−0.13 to 0.11)
Range of motion at 6 months	109.8 ± 15.1	107.5 ± 15.3	0.032	0.13 (0.01 to 0.26)
*Patient Satisfaction*				
% Satisfied	95.6%	90.8%	0.003	—
*Tibiofemoral angle*				
Preoperative tibiofemoral angle	5.8° ± 4.3°	6.5° ± 4.5°	0.017	0.16 (0.04–0.28)
Postoperative tibiofemoral angle	4.5° ± 2.1°	4.2° ± 1.9°	0.030	0.15 (0.03–0.27)

^*^Oxford Knee Score (OKS) outcomes according to the original scoring convention, with scores ranging from 12 to 60, where lower scores indicate better knee function. Positive change scores represent improvement and were calculated as baseline OKS minus postoperative OKS

### 2 Year postoperative outcomes

At 24 months, excellent outcomes were sustained in both groups. KA demonstrated significantly higher postoperative Knee Society Knee Scores (KSKS) (89.2 versus 86.3, *p* < 0.001, *d* = 0.26, 95% CI 0.14 to 0.38) and Knee Society Function Scores (KSFS) (77.6 versus 75.6, *p* = 0.022, *d* = 0.15, 95% CI 0.03 to 0.27), although change-from-baseline scores (KSKS change *d* = 0.11, 95% CI −0.01 to 0.23; KSFS change *d* = 0.06, 95% CI −0.06 to 0.18) and MCID attainment for both measures were comparable. Oxford Knee Scores (*d* = −0.07, 95% CI −0.19 to 0.05), change scores (*d* = −0.03, 95% CI −0.15 to 0.09) and MCID attainment were also similar between groups.

KA maintained superior range of motion at 2 years, with greater end-range flexion (116.6° versus 114.0°, *p* < 0.001, *d* = 0.15, 95% CI 0.03 to 0.27) and greater arc of motion (115.7° versus 113.1°, *p* = 0.001, *d* = 0.14, 95% CI 0.02 to 0.27), while extension remained comparable (*d* = −0.02, 95% CI −0.14 to 0.10). Among SF-36 domains, KA demonstrated significantly higher physical functioning scores (75.7 versus 72.0, *p* < 0.001, *d* = 0.21, 95% CI 0.09 to 0.34), whereas all other SF-36 domains, including bodily pain (*d* = 0.04, 95% CI −0.08 to 0.16), PCS (*d* = 0.09, 95% CI −0.04 to 0.21) and MCS (*d* = −0.01, 95% CI −0.13 to 0.11), showed no significant between-group differences. Patient satisfaction remained high and comparable between groups (95.3% versus 95.7%, *p* = 0.838) (Table 4). Secondary SF-36 subscale outcomes at 2 years are reported in Supplementary Table 2.

**Table 4:** 24-Month PROMs and ROM outcomes

	KA	MA	*p*-value	Cohen’s *d* (95% CI)
*Knee Society Knee Score (KSKS)*				
Score at 2 years	89.2 ± 10.3	86.3 ± 11.7	< 0.001	0.26 (0.14 to 0.38)
Change from baseline	51.8 ± 19.2	49.5 ± 20.8	0.089	0.11 (−0.01 to 0.23)
MCID attained (≥ 6.4), %	98.1%	96.1%	0.093	—
*Knee Society Function Score (KSFS)*				
Score at 2 years	77.6 ± 18.7	75.6 ± 17.8	0.022	0.15 (0.03 to 0.27)
Change from baseline	24.3 ± 21.4	22.9 ± 20.1	0.164	0.06 (−0.06 to 0.18)
MCID attained (≥ 5.9), %	81.1%	80.4%	0.828	—
Oxford Knee Score (OKS)^*^				
Score at 2 years	17.7 ± 4.5	18.1 ± 4.4	0.053	−0.07 (−0.19 to 0.05)
Change from baseline	17.2 ± 7.9	17.3 ± 7.9	0.961	−0.03 (−0.15 to 0.09)
MCID attained (≥ 5.0), %	95.9%	95.5%	0.890	—
*SF-36 Physical Function*				
Score at 2 years	75.7 ± 20.3	72.0 ± 19.3	< 0.001	0.21 (0.09 to 0.34)
Change from baseline	34.5 ± 26.1	32.7 ± 23.2	0.431	0.08 (−0.04 to 0.20)
MCID attained (≥ 11.6), %	80.7%	81.5%	0.792	—
*SF-36 Role Physical*				
Score at 2 years	75.6 ± 37.3	77.4 ± 35.1	0.921	−0.04 (−0.16 to 0.08)
Change from baseline	57.1 ± 46.8	61.1 ± 45.1	0.200	−0.10 (−0.21 to 0.03)
MCID attained (≥ 11.7), %	73.5%	78.5%	0.068	—
*SF-36 Bodily Pain*				
Score at 2 years	73.3 ± 24.6	72.6 ± 25.3	0.747	0.04 (−0.08 to 0.16)
Change from baseline	39.8 ± 27.7	40.1 ± 28.2	0.682	−0.01 (−0.13 to 0.11)
MCID attained (≥ 16.9), %	81.1%	79.6%	0.611	—
*SF-36 Physical Component Summary (PCS)*				
Score at 2 years	49.7 ± 8.0	49.1 ± 8.7	0.110	0.09 (−0.04 to 0.21)
Change from baseline	16.9 ± 11.4	17.0 ± 11.3	0.696	−0.02 (−0.14 to 0.10)
MCID attained (≥ 10.0), %	72.8%	74.4%	0.601	—
*SF-36 Mental Component Summary (MCS)*				
Score at 2 years	57.0 ± 9.7	57.1 ± 9.2	0.767	−0.01 (−0.13 to 0.11)
Change from baseline	4.7 ± 11.7	5.1 ± 10.8	0.247	−0.07 (−0.18 to 0.06)
MCID attained (≥ 10.0), %	29.1%	31.2%	0.488	—
*Range of Motion*				
Flexion at 2 years	116.6 ± 13.5	114.0 ± 12.9	< 0.001	0.15 (0.03 to 0.27)
Extension at 2 years	0.8 ± 4.3	1.0 ± 4.2	0.919	−0.02 (−0.14 to 0.10)
Range of motion at 2 years	115.7 ± 14.9	113.1 ± 14.4	0.001	0.14 (0.02 to 0.27)
*Patient satisfaction*				
% Satisfied	95.3%	95.7%	0.838	—

^*^Oxford Knee Score (OKS) outcomes according to the original scoring convention, with scores ranging from 12 to 60, where lower scores indicate better knee function. Positive change scores represent improvement and were calculated as baseline OKS minus postoperative OKS

### Complication rates

**Fig. 2:**
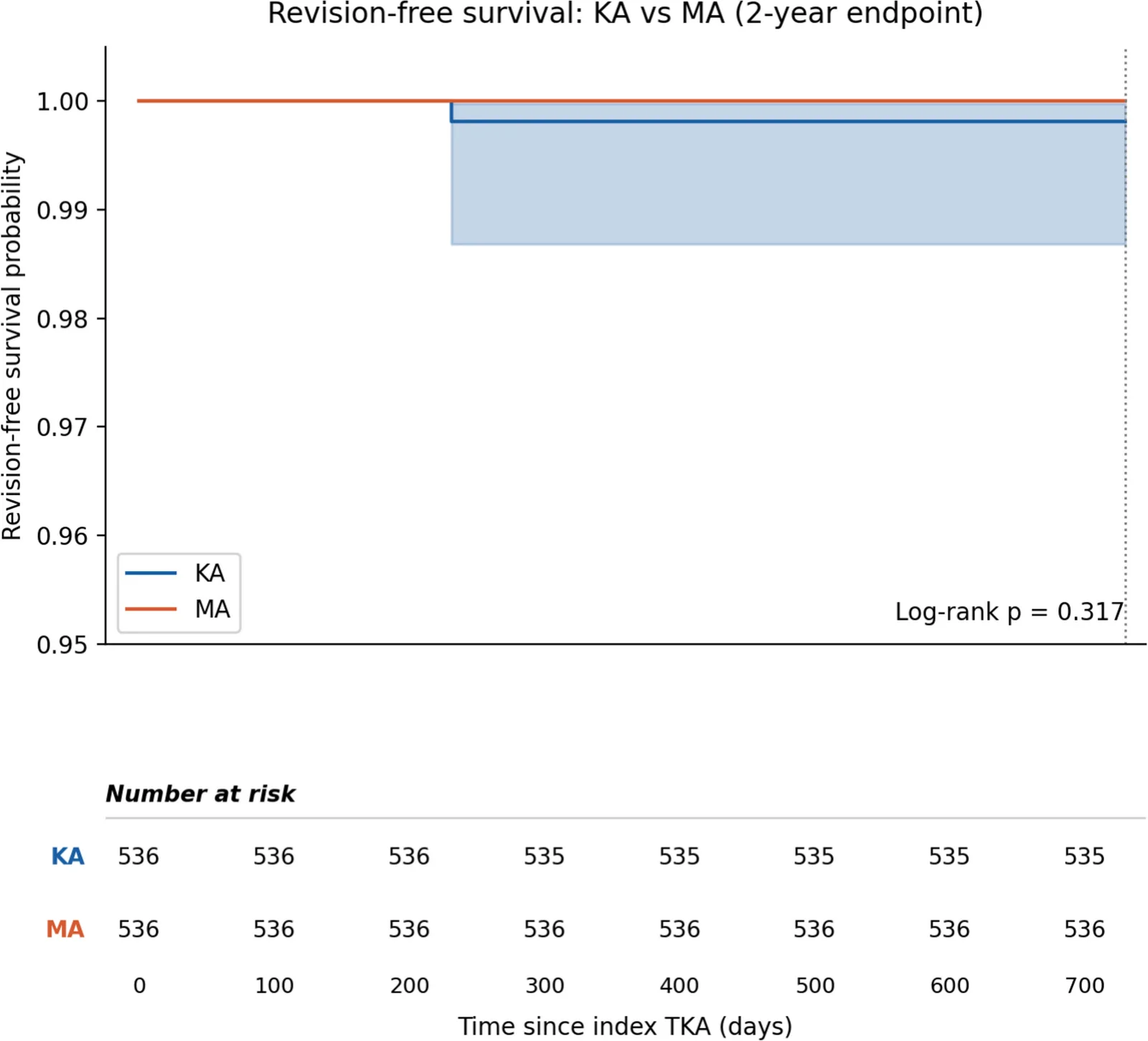
Revision-free survival (Kaplan–Meier, 2-year endpoint)

Within the propensity score–matched cohort, complications were infrequent and comparable between groups, with 6 events (1.1%) in the KA-TKA group and 8 (1.5%) in the MA-TKA group (*p* = 0.789). In the KA-TKA group, five patients (0.9%) required MUA for postoperative stiffness and one (0.2%) underwent arthrotomy and polyethylene insert exchange for recurvatum at 231 days. In the MA-TKA group, five patients (0.9%) required MUA and three (0.6%) sustained periprosthetic fractures, managed with retrograde femoral nailing, conservative bracing or ORIF. No prosthetic joint infection (PJI) or periprosthetic fractures occurred in the KA-TKA group, and no insert exchanges or mechanical revisions occurred in the MA-TKA group (Table 5). Kaplan–Meier analysis demonstrated comparable two-year revision-free survival (KA 99.8% versus MA 100.0%, log-rank *p* = 0.317) (Fig. 2).

**Table 5:** Complication rates

Complication	Management	KA (*N* = 536)	MA (*N* = 536)
Stiffness	Manipulation under anaesthesia	5 (0.9%)	5 (0.9%)
Instability or recurvatum	Arthrotomy + polyethylene insert exchange	1 (0.2%)	0
Periprosthetic fracture	One was managed with retrograde femoral nail insertion, one was managed with immobilisation and bracing and one underwent open reduction and internal fixation (ORIF)	0	3 (0.6%)
Periprosthetic joint infection	—	0	0
Any complication	—	6 (1.1%)	8 (1.5%)
All-cause reoperation	—	6 (1.1%)	7 (1.3%)
All-cause revision	—	1 (0.2%)	0 (0.0%)

## Discussion

This study demonstrates an association between kinematic alignment TKA and modestly higher postoperative scores in selected functional domains, including range of motion and KSKS, alongside no significant difference in safety profile or revision-free survival. According to a systematic review by Rivière et al., KA is associated with accelerated early recovery and superior short-term functional outcomes compared with MA [26]. These benefits are hypothesised to result from the restoration of native joint-line geometry and ligament tension, reduced need for soft-tissue releases and more physiologic kinematics that facilitate pain reduction and improved gait. However, the magnitude and durability of the functional advantage vary across studies and patient subgroups, and concerns remain about load distribution and implant longevity in patients with severe preoperative deformity. Therefore, careful patient selection and long-term comparative data are required to define the subpopulations that derive the greatest net benefit from KA.

Our findings are broadly consistent with a meta-analysis demonstrating greater short-term outcomes with KA-TKA compared with MA-TKA, including improved Knee Society scores and greater postoperative knee flexion [27]. Similarly, Yeo et al. reported comparable clinical and radiographic outcomes between robotic MA and KA strategies at a mean follow-up of eight years [28]. Our findings are broadly consistent with this literature. In our cohort, KA demonstrated greater early postoperative outcomes, with higher Knee Society Knee Scores, greater postoperative flexion and range of motion, improved physical functioning and higher patient satisfaction at 6 months. These functional advantages persisted at 24 months, with KA maintaining greater Knee Society Knee and Function Scores together with sustained improvements in range of motion and physical functioning. Although several outcomes demonstrated statistically significant between-group differences, the absolute magnitude of these differences was modest. Importantly, the majority of statistically significant differences were not accompanied by significant differences in MCID attainment. At both 6 months and 2 years, MCID attainment for KSKS, KSFS, OKS and SF-36 Physical Function was comparable between groups. Conversely, at 6 months, MA demonstrated higher MCID attainment for SF-36 Role Physical and Bodily Pain, despite these differences being modest in absolute terms. Taken together, these findings suggest that KA may provide measurable improvements in certain objective and functional outcomes, but that the magnitude of these differences may be insufficient to consistently translate into clinically perceptible patient-level benefits. Given the multiple outcomes and follow-up time points evaluated, the possibility of type I error due to multiple comparisons cannot be excluded. No formal multiplicity adjustment was performed; therefore, significant findings should be interpreted cautiously alongside effect sizes and MCID thresholds.

Use of patient-specific instrumentation to perform kinematically aligned TKA without limiting preoperative deformity did not compromise implant survival, annual revision rates or functional outcomes over a 10-year period [29]. Similarly, at a mean follow-up of 6.3 years, residual varus of the tibial component, knee or limb after KA-TKA was not associated with reduced implant survival or poorer function, supporting KA as a valid alternative to mechanical alignment for primary TKA [30].

Complication rates were low and comparable between KA-TKA and MA-TKA (1.1% versus 1.5%), with similar rates of MUA for postoperative stiffness (0.9% versus 0.9%). Periprosthetic fractures occurred only in the MA-TKA group (0.6%), while the KA-TKA group had one insert exchange for recurvatum. Two-year revision-free survival was similarly high in both groups (99.8% versus 100.0%, log-rank *p* = 0.317), suggesting that no significant difference in early complication or revision rates was observed between KA-TKA and MA-TKA.

Key strengths of this study include the application of propensity score matching to minimise confounding between groups, and the direct comparison of two alignment performed exclusively by a single experienced surgeon within a single institution, with perioperative and rehabilitation protocols standardised within each operative era. We note that the institutional ERAS pathway was introduced in 2021 and therefore applied only to the later portion of the KA cohort; its potential contribution to the observed differences has been acknowledged in the Limitations. The systematic collection of multiple validated patient-reported measures and functional outcome measures by trained physiotherapists further reduces the risk of assessor bias.

Several limitations warrant acknowledgement. The retrospective, non-concurrent design is an important limitation of this study. The MA-TKA cohort was recruited between 2010 and 2014 and the KA-TKA cohort between 2018 and 2023. Although propensity score matching was applied to balance measured baseline characteristics, it cannot fully account for secular trends in perioperative practice, surgical experience, patient expectations or institutional culture over this interval. Notably, the institutional enhanced recovery after surgery (ERAS) pathway for TKA was introduced only in 2021 and therefore did not apply to the earlier MA-TKA cohort and was relevant only to the later portion of the KA-TKA cohort. Nevertheless, the potential influence of other unmeasured temporal changes cannot be excluded. Furthermore, propensity score matching cannot account for unmeasured temporal confounding arising from the non-concurrent study periods. Therefore, the observed differences should be interpreted as associations rather than causal effects of alignment strategy.

Also, while the 2-year follow-up period permits assessment of early to mid-term functional outcomes, it is insufficient to draw conclusions regarding long-term implant survivorship in the KA-TKA cohort, and extended follow-up will be necessary to address this question. Furthermore, knee alignment readings were derived using a goniometer by a trained physiotherapist. The current gold standard measurement for varus/valgus alignment is to use the mechanical axis of the hip-knee-angle using a full-limb radiograph. However, studies have shown high correlation between the goniometer and mechanical axis for knee alignment, making the goniometer a valid alternative [24]. Lastly, The loss to follow-up differed between groups (KA: 293/885, 33.1%; MA: 148/1,017, 14.6%), likely reflecting the more recent operative dates of the KA cohort rather than a systematic difference in outcomes; however, this differential attrition represents a potential source of selection bias that cannot be fully excluded.

## Conclusions

This large-scale, propensity score matched, single-surgeon study finds that KA-TKA is associated with statistically higher scores in selected functional domains—including postoperative range of motion and KSKS—and modestly higher physical functioning at 6 months and 2 years. However, the magnitude of these differences was modest, MCID attainment and patient satisfaction at 2 years were largely comparable between groups, and KA was not associated with increased complication or revision rates at 2 years. Kaplan–Meier analysis demonstrated no significant difference in 2-year revision-free survival. Given the non-concurrent cohort design, the observed differences may partly reflect secular trends in perioperative care rather than alignment strategy alone. Prospective comparative data with longer follow-up are required to confirm these associations and determine the durability of implant survivorship under kinematic alignment principles.

## Supplementary Information

Supplementary Material 1.

## Data Availability

The datasets that support the findings of this study were derived from the institutional total knee arthroplasty registry. Restrictions apply to the availability of these data due to patient confidentiality and institutional data governance policies; therefore, the data are not publicly available. Data may be made available from the corresponding author upon reasonable request and with appropriate institutional approval.
