# Supplementary material for: Two-year functional outcomes of kinematic versus mechanical alignment in primary total knee arthroplasty: a single-surgeon propensity-matched study in an Asian population
Source: Knee Surg Relat Res. 2026 Nov 13;38:57. doi: 10.1186/s43019-026-00359-9 (PMC13769187; doi:10.1186/s43019-026-00359-9)
Supplement: Supplementary file 1 [file 43019_2026_359_MOESM1_ESM.docx]

**Supplementary Table 1: Secondary SF-36 subscale outcomes at 6 months**

| **SF-36 General Health** | | | | |
| --- | --- | --- | --- | --- |
| Score at 6 months | 73.2 ± 20.0 | 74.0 ± 19.0 | 0.752 | -0.02 (-0.14 to 0.10) |
| Change from baseline | 4.5 ± 20.7 | 6.0 ± 20.5 | 0.202 | -0.06 (-0.18 to 0.06) |
| MCID attained (≥0.9), % | 53.9% | 55.4% | 0.655 | – |
| **SF-36 Vitality** | | | | |
| Score at 6 months | 75.8 ± 18.8 | 76.6 ± 19.4 | 0.292 | -0.03 (-0.15 to 0.09) |
| Change from baseline | 4.8 ± 22.2 | 6.0 ± 22.7 | 0.110 | -0.07 (-0.19 to 0.05) |
| MCID attained (≥3.9), % | 49.0% | 50.9% | 0.561 | – |
| **SF-36 Social Function** | | | | |
| Score at 6 months | 87.6 ± 23.9 | 87.0 ± 26.2 | 0.608 | 0.05 (-0.07 to 0.17) |
| Change from baseline | 31.9 ± 39.9 | 34.3 ± 38.5 | 0.297 | -0.06 (-0.18 to 0.06) |
| MCID attained (≥11.7), % | 62.1% | 66.0% | 0.206 | – |
| **SF-36 Role Emotional** | | | | |
| Score at 6 months | 96.7 ± 16.1 | 96.8 ± 16.7 | 0.374 | 0.03 (-0.09 to 0.15) |
| Change from baseline | 5.0 ± 29.2 | 5.5 ± 29.7 | 0.370 | -0.02 (-0.14 to 0.10) |
| MCID attained (≥7.8), % | 8.7% | 9.3% | 0.785 | – |
| **SF-36 Mental Health** | | | | |
| Score at 6 months | 86.4 ± 13.1 | 86.4 ± 14.0 | 0.383 | -0.00 (-0.12 to 0.12) |
| Change from baseline | 5.7 ± 17.5 | 6.3 ± 18.1 | 0.224 | -0.04 (-0.17 to 0.08) |
| MCID attained (≥0.3), % | 49.2% | 54.5% | 0.093 | – |

**Supplementary Table 2: Secondary SF-36 subscale outcomes at 2 years**

| **SF-36 General Health** | | | | |
| --- | --- | --- | --- | --- |
| Score at 2 years | 73.7 ± 20.5 | 73.0 ± 19.6 | 0.362 | 0.03 (-0.09 to 0.15) |
| Change from baseline | 5.1 ± 23.0 | 4.9 ± 21.5 | 0.957 | -0.01 (-0.13 to 0.11) |
| MCID attained (≥0.9), % | 57.2% | 54.4% | 0.384 | – |
| **SF-36 Vitality** | | | | |
| Score at 2 years | 77.0 ± 18.1 | 77.8 ± 18.6 | 0.297 | -0.05 (-0.17 to 0.07) |
| Change from baseline | 6.0 ± 22.5 | 7.2 ± 22.5 | 0.371 | -0.09 (-0.21 to 0.03) |
| MCID attained (≥3.9), % | 52.3% | 50.5% | 0.580 | – |
| **SF-36 Social Function** | | | | |
| Score at 2 years | 92.7 ± 19.1 | 91.3 ± 22.2 | 0.915 | 0.07 (-0.06 to 0.18) |
| Change from baseline | 37.0 ± 37.9 | 38.6 ± 39.1 | 0.317 | -0.06 (-0.18 to 0.06) |
| MCID attained (≥11.7), % | 64.9% | 67.9% | 0.342 | – |
| **SF-36 Role Emotional** | | | | |
| Score at 2 years | 98.1 ± 12.9 | 96.0 ± 18.9 | 0.072 | 0.10 (-0.03 to 0.22) |
| Change from baseline | 6.3 ± 28.5 | 4.7 ± 30.0 | 0.569 | 0.01 (-0.11 to 0.13) |
| MCID attained (≥7.8), % | 8.6% | 8.6% | 1.000 | – |
| **SF-36 Mental Health** | | | | |
| Score at 2 years | 86.6 ± 13.7 | 86.8 ± 13.4 | 0.995 | 0.00 (-0.12 to 0.12) |
| Change from baseline | 5.9 ± 18.0 | 6.7 ± 18.4 | 0.342 | -0.05 (-0.17 to 0.07) |
| MCID attained (≥0.3), % | 52.9% | 56.8% | 0.221 | – |
